# P23 A case of partial restoration of vision in a patient with Wegener's granulomatosis and acute proptosis without signs of pseudotumor of orbit

**DOI:** 10.1093/rap/rkab068.022

**Published:** 2021-10-19

**Authors:** Oksana Golovina, Anna Torgashina, Evgeniya Sokol

**Affiliations:** V.A. Nasonova Research Institute of Rheumatology, Moscow, Russian Federation

## Abstract

**Case report - Introduction:**

Ophthalmologic manifestations occur in half of the patients with Wegener's granulomatosis and about 8% result in a complete loss of vision. Proptosis is a specific manifestation of Wegener's granulomatosis and often leads to a complete loss of vision due to ischemia of the optic nerve. In the literature there are rare cases of proptosis in these patients which are not accompanied by pseudotumor development. We present the case of a patient with histologically confirmed Wegener's granulomatosis who developed acute proptosis with a complete vision loss over the course of a week and a partial restoration of vision during therapy.

**Case report - Case description:**

In March 2020 a 60-year-old man was diagnosed with Wegener's granulomatosis (based on lung biopsy) with involvement of lung (infiltrates with a cavity of destruction), upper respiratory tract (ethmoiditis, sphenoiditis, bloody crusts in the nose), ANCA-MPO+. 40mg of prednisone was prescribedwith a gradual decrease to 10mg.

In the summer of 2020, the patient had a saddle-shaped deformity of the nose.

In October he first noted a gradual decrease and then a complete loss of vision in the right eye, accompanied by temporary proptosis and headaches.

In November 2020 CT scan of the orbits showed no signs of pathology. Intravenous therapy with methylprednisolone 2250mg in total and cyclophosphamide 800mg was carried out, the dose of prednisolone per os was increased to 15mg.

After 2 weeks, the patient developed an acute complete vision loss in the left eye, accompanied by proptosis, pain in the orbit, headaches and ophthalmoplegia. He was admitted to ophthalmological hospital; glaucoma was ruled out. A spectral domain optical coherence tomography of the left eye was performed: the thickness of the layer of nerve fibres was within normal limits, the macular profile, the differentiation of all layers of the retina was preserved.

A week after a complete loss of vision, the patient was transferred to Nasonova Research Institute of Rheumatology where therapy with rituximab (1g + 1g with an interval of 2 weeks), intravenous therapy with methylprednisolone 2750mg in total, anticoagulant, ethylmethylhydroxypyridine succinate, eradication therapy were carried out. During treatment, the patient noted a gradual partial restoration of vision (the ability to see large and medium objects with the left eye), disappearance of pain, and a decrease of proptosis. On MRI 2 days after the start of therapy, signs of exudative ethmoidite and sphenoidite and chronical mastoidite were revealed.

**Case report - Discussion:**

Proptosis is one of the most common and specific ophthalmic manifestations of Wegener's granulomatosis. Proptosis is most often caused by a pseudotumor of the orbit; however, in this patient the genesis of proptosis is not clear because no signs of pseudotumor were detected on CT before the start of therapy or on MRI 2 days after therapy. It is possible that this patient had retrobulbar cellulitis with compressive ischemia of the optic nerve, single descriptions of which are found in the literature. However, its bacterial genesis was doubtful because there wasn't a change for the worse after 7 days of absence of antibiotic therapy. Alternatively, this patient could have developed Tolosa—Hunt syndrome or cavernous sinus thrombosis against the background of exudative sinusitis which can also manifest with similar symptoms. However, the fact that the symptoms developed during therapy of hormones per os (the dose remained stable) is contradicted by the development of the Tolosa—Hunt syndrome. During decrease in hormones for the observation period (half a year) relapses of these symptoms weren’t noted. CT and MRI showed no signs of cavernous thrombosis or Tolosa—Hunt syndrome. However, all the above-mentioned arguments are just our speculation because, due to the urgency of the situation and technical reasons, we could not perform MRI of the orbit before the start of therapy.

Despite the large number of questions regarding the cause of vision loss in this patient, we decided to describe this case, since the therapy performed in this patient was very effective and led to a significant restoration of vision, even despite the fact it began a week later from the occurrence of vision loss. Perhaps in the future, our experience could be useful for other doctors who come across a similar difficult diagnostic situation.

**Case report - Key learning points:**

Despite the fact that this patient had an established reliable diagnosis of Wegener's granulomatosis, this situation has left many questions for both our rheumatologists and ophthalmologists. This patient had many risk factors for an acute vision loss, both depending on the underlying disease and the risks of developing concomitant pathology, so we can’t make a final decision on the genesis of his symptoms. However, such difficult patients can be found by every ophthalmologist or rheumatologist, thus it is important to accumulate knowledge about their treatment. In this regard, we would like to present this case and share this successful experience of treatment with our colleagues.

